# Genome Sequences of *Streptomyces* Bacteriophages Fabian, FlowerPower, Geostin, RetrieverFever, and Vorvolakos

**DOI:** 10.1128/mra.00998-22

**Published:** 2022-11-03

**Authors:** Faith Davis, Baily Kakacek, Delaram Doorandish, Tamar Goldwasser, Neyiasuo Agboha, 2021 UMBC Phage Hunters, STEM BUILD at UMBC Cohort 6, Steven M. Caruso, Ivan Erill

**Affiliations:** a Department of Biological Sciences, University of Maryland, Baltimore County, Baltimore, Maryland, USA; b College of Natural and Mathematical Sciences, University of Maryland, Baltimore County, Baltimore, Maryland, USA; Portland State University

## Abstract

This paper reports the genome sequences of five bacteriophages that were isolated using Streptomyces scabiei. Phages Fabian, FlowerPower, Geostin, RetrieverFever, and Vorvolakos were assigned to actinobacteriophage cluster BF based on shared gene content, with each phage containing between 16 and 21 tRNA genes.

## ANNOUNCEMENT

Five bacteriophages were isolated using the phytopathogenic bacterium Streptomyces scabiei RL-34 and annotated as part of the Science Education Alliance-Phage Hunters Advancing Genomics and Evolutionary Science (SEA-PHAGES) program (1).

**FIG 1:**
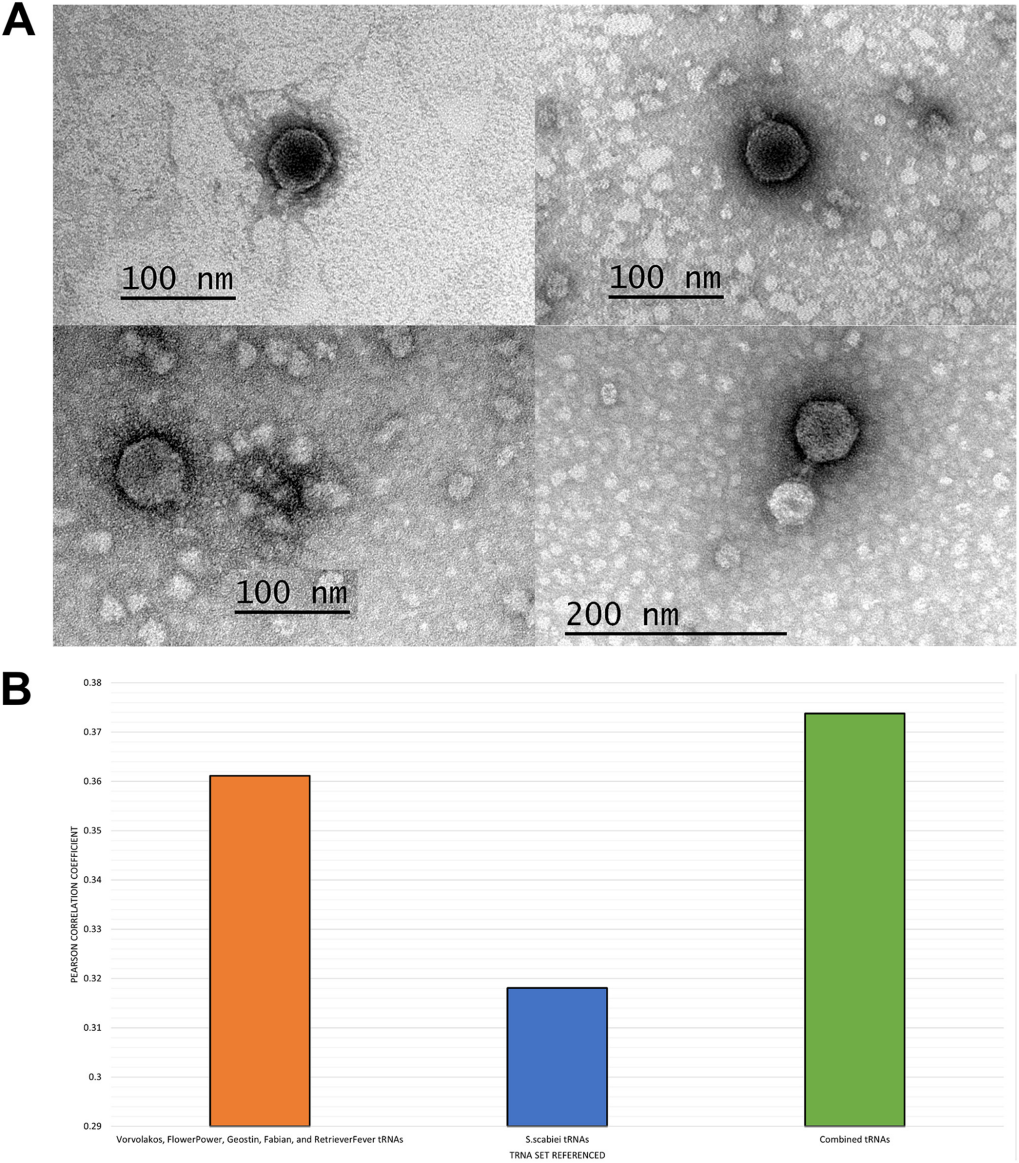
Characterization of phages and tRNA genes. (A) Representative transmission electron microscopy images of the phages described in this paper. (Top left) Fabian. (Top right) FlowerPower. (Bottom left) Geostin. (Bottom right) RetrieverFever. (B) Pearson correlation coefficients for correlations between codons recognized by the products of tRNA genes included in *Streptomyces* BF cluster phage genomes, the S. scabiei genome, or the combined phage and S. scabiei genomes and the codons present in the gene coding for the MCP in each phage. Codon usage correlates better with the combined tRNA pool.

Phages were isolated from soil samples collected across Maryland, USA (Table 1), using standard methods (2). Briefly, soil samples were suspended in phage buffer (10 mM Tris [pH 7.5], 10 mM MgSO_4_, 1 mM CaCl_2_, 68.5 mM NaCl). Suspensions were centrifuged to pellet soil, and the supernatant was filtered (0.22-μm filter). The filtrate was then plated in tryptic soy soft agar (BD) with S. scabiei RL-34 on nutrient agar plates (BD Difco) supplemented with 10 mM MgCl_2_, 8 mM Ca(NO_3_)_2_, and 0.5% glucose. Incubation at 30°C for 1 to 2 days yielded bacteriophages Fabian, FlowerPower, Geostin, RetrieverFever, and Vorvolakos. A minimum of three rounds of plaque purification were performed for each phage. After 24 to 48 h at 30°C on S. scabiei, all phages except Vorvolakos formed round 1.5- to 5-mm-diameter plaques with turbid halos; Vorvolakos formed clear plaques. Negative-staining transmission electron microscopy revealed these phages to be podoviruses, with capsid widths ranging from 50 to 61 nm (Fig. 1A).

**TABLE 1:** Properties of five cluster BF *Streptomyces* phage genomes

Phage	Sample collection location coordinates	No. of genes encoding proteins with assigned functions	No. of tRNA genes	Genome length (bp)	No. of reads	Sequencing coverage (×)	GenBank accession no.	SRA accession no.
Fabian	39.25281N, 76.851488W	18	18	46,505	649,913	2,003	MN284894	SRX14814645
FlowerPower	39.256894N, 76.714128W	13	21	46,133	1,596,259	5,190	MH155868	SRX14814646
Geostin	39.140777N, 76.528469W	16	16	46,175	537,680	1,661	MN119376	SRX14814647
RetrieverFever	39.23N, 77.24W	18	21	46,175	898,381	2,778	MZ622160	SRX14814649
Vorvolakos	39.58972N, 76.28048W	20	21	46,175	4,523,515	13,943	ON108647	SRX14485104

Phage DNA was isolated using the Promega Wizard DNA purification system on a freshly prepared lysate as described previously (3). Sequencing of Fabian, Geostin, RetrieverFever, and Vorvolakos was completed by the Pittsburgh Bacteriophage Institute; sequencing of FlowerPower was completed by the North Carolina State University Genomic Sciences Laboratory. All sequencing was performed with the Illumina MiSeq (v3 reagents) sequencing platform using the NEBNext Ultra II library preparation kit and 150-base single-end reads. Raw sequencing reads were assembled using Newbler v2.9 or the CLC Genomics Workbench next-generation sequencing (NGS) *de novo* assembler v6 with default settings. Genome completeness and termini were determined using Consed v29 (4, 5). All phages were found to have linear chromosomes with 253-bp direct terminal repeats and an average GC content of 60.07% (standard deviation, ±0.44%) (Table 1). Based on gene content similarity, the Actinobacteriophage Database (phagesDB) assigned these phages to the BF cluster (6–8).

Genome annotation was completed using DNA Master v5.23.6 (9) embedded with Glimmer v3.02b (10) and GeneMark v4.28 (11), with manual refinement using parameters such as proximity and directionality with respect to protein-encoding sequences, the presence of putative ribosome binding sites, and sequence similarity to previously annotated genes (12). Protein-encoding genes were functionally annotated using BLASTp (13), Phamerator v3.0 (14), and HHPred v57c87 (15, 16). The numbers of genes encoding proteins with known functions ranged from 13 to 20 (Table 1). The numbers of tRNA genes found using ARAGORN v1.2.41 ranged from 16 to 21 (17). All cluster BF phages contain 15 to 20 tRNAs in a single genome segment. The numbers of codons in their major capsid protein (MCP) gene sequences, as well as the numbers of host and phage tRNAs recognizing each codon, were tabulated, and their correlation was analyzed. Our data support the observation that phage tRNAs match the codon usage for the MCPs better than do host tRNAs (Fig. 1B) (18).

### Data availability.

GenBank nucleotide record and Sequence Read Archive (SRA) accession numbers for all genomes reported in this work are provided in Table 1.
